# Purinergic Signalling in Group A *Streptococcus* Pathogenesis

**DOI:** 10.3389/fimmu.2022.872053

**Published:** 2022-03-29

**Authors:** T. B-D McEwan, M. L. Sanderson-Smith, R. Sluyter

**Affiliations:** ^1^ Illawarra Health and Medical Research Institute, Wollongong, NSW, Australia; ^2^ Molecular Horizons and School of Chemistry and Molecular Bioscience, University of Wollongong, Wollongong, NSW, Australia

**Keywords:** *Streptococcus pyogenes*, purinergic receptor, A_2A_ receptor, ectonucleotidase, P2X7 receptor, LL-37, innate immunity, virulence factor

## Introduction

*Streptococcus pyogenes* (Group A *Streptococcus*; GAS*)* is a human specific bacterial pathogen, recognised globally as one of the top ten leading causes of infectious disease mortality (1). It is responsible for causing a wide range of pathologies from superficial skin infections to invasive disease including necrotising fasciitis (1). Post-*streptococcal* sequelae, such as rheumatic heart disease, are severe complications that develop in response to repeated infections and are largely responsible for the global GAS-related death toll surpassing 500,000 fatalities annually (2). GAS expresses various virulence factors to subvert the host innate immune system, which is central to controlling GAS infection (3). Since current treatment strategies are failing (4), there is a need to further examine the (dys)regulation of host immune networks underlying GAS pathophysiology and identify novel therapeutic targets to arrest disease progression.

Purinergic signalling is an important immunomodulatory network in health and disease. In response to bacterial infections, damaged cells release adenosine 5′-triphosphate (ATP) and other nucleotides that can activate ionotropic P2X receptors and metabotropic P2Y receptors to promote a pro-inflammatory phenotype through cytokine production and immune cell recruitment. Cell surface ectonucleotidases, including CD39 and CD73, help arrest this pro-inflammatory response by hydrolysing exogenous ATP and adenosine 5’-diphosphate (ADP) to adenosine to restrict P2 receptor activation. This in turn stimulates metabotropic P1 (adenosine) receptors to promote an anti-inflammatory phenotype in immune and other cell types (5).

Several bacterial species can modulate the purinergic signalling network to enhance infection (6). Furthermore, nucleotides can modulate immune cell functions such as mTOR signalling and mitochondrial activation in neutrophils to regulate the chemotaxis of these cells (7, 8), which have key roles in regulating GAS and other infections (9). Concurrent with purinergic receptor activation, a hyperinflammatory response is characteristic of invasive GAS infection (10), but current understanding of purinergic signalling mechanisms in GAS pathogenesis and host immunity remains limited. This opinion article provides a brief overview of current research data available on purinergic signalling in GAS infection and the potential of this signalling system as a therapeutic target in GAS infection.

## GAS Virulence Factors as a Nexus to Purinergic Signalling

A current priority in GAS research is understanding the molecular mechanisms associated with disease progression in order to establish potential therapeutic targets. Underlying invasive GAS pathogenesis is the ability of this bacterium to subvert and exploit the host innate immune response, which is partially attributed to virulence factor expression (3). A major conserved GAS virulence factor is the antigenically diverse M protein with M1-expressing GAS strains reported to have a high global prevalence (11). As explained below, several GAS virulence factors have been associated with purinergic signalling pathways, indirectly supporting a role for purinergic signalling in GAS infection.

## Extracellular Nucleotide Metabolism as a Mode of Immune Evasion by GAS

Secreted virulence factors are commonly associated with invasive inflammation and pathology (10). *Streptococcal* 5′-nucleotidase A (S5nA) is a secreted virulence factor and bacterial ectonucleotidase first identified in M1-expressing GAS (12). Similar to the mammalian ectonucleotidases that aid in immune regulation (5), S5nA hydrolyses extracellular nucleotides to adenosine, with a rank order of deoxygenated adenosine 5’-monophosphate (AMP) > AMP > ADP, and no effect on ATP (12). Although this study provided evidence of increased production of Sn5A during invasive GAS infection through sera analysis from infected patients, a clear role for this virulence factor over the course of disease is yet to be elucidated but may relate to either adenosine production and/or nucleotide reduction. A detailed description of bacterial ectonucleotidases and roles in infection is beyond the scope of this article, but readers are directed to a recent review on this topic (13).

**Figure 1:**
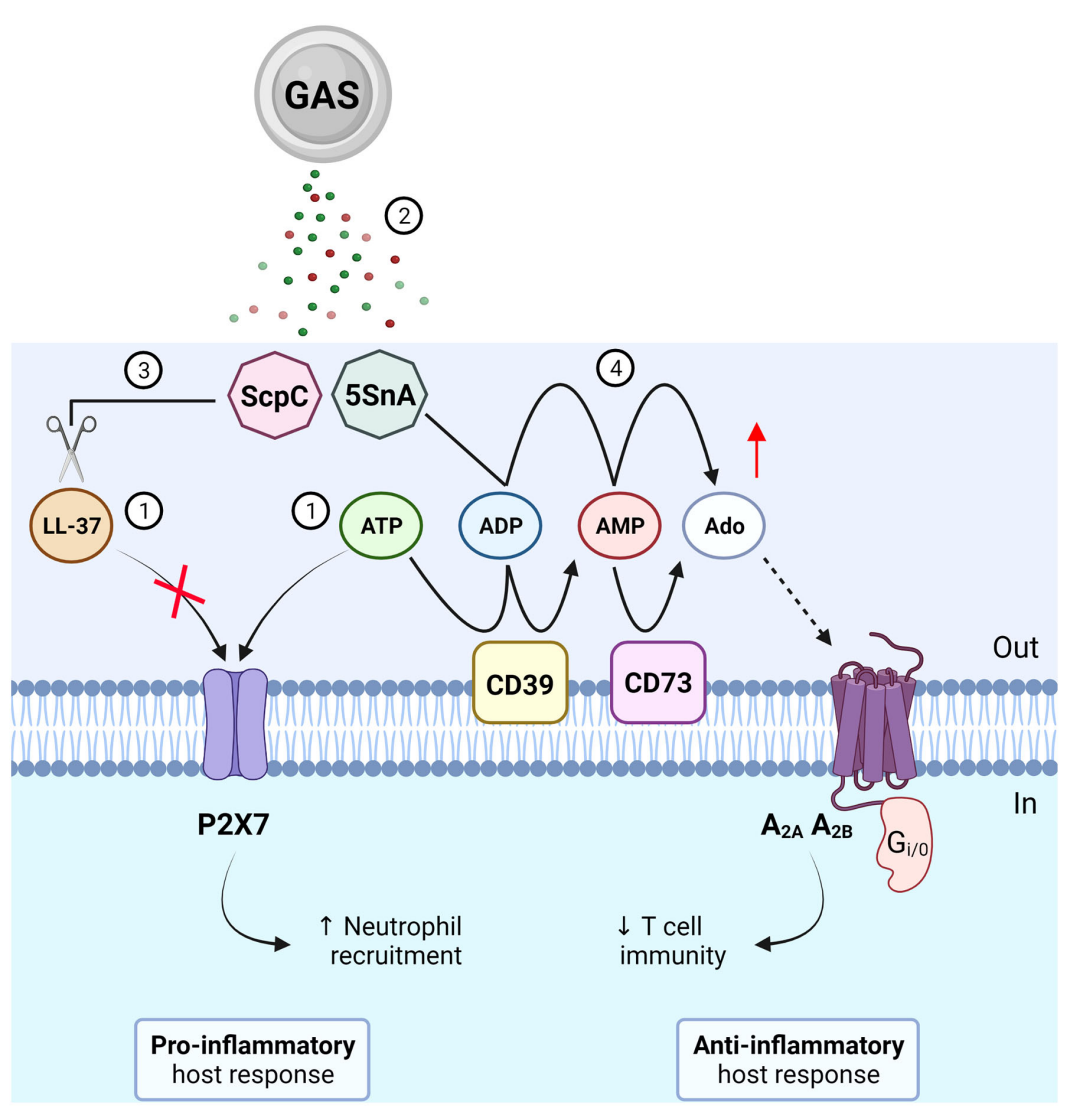
Roles of purinergic signalling in Group A *Streptococcus* (GAS) immunity and evasion. (1) GAS causes the release of LL-37 and ATP, which can activate P2X7 to promote neutrophil recruitment and protection from GAS infection. (2) GAS secretes virulence factors, ScpC and streptococcal 5’-ectonucleotidase A (5SnA). (3) ScpC can cleave LL-37 to prevent P2X7 activation and promote immune evasion including reduced neutrophil recruitment. (4) 5SnA can hydrolyse ADP and AMP to increase extracellular adenosine (Ado), mimicking the action of host ectonucleotidases (CD39 and CD73), to activate P1 receptors (A_2A_ and A_2B_) and potentially restrain T cell immunity and promote immune evasion. Created with BioRender.com.

Extracellular adenosine production is advantageous for bacterial survival as it suppresses the host inflammatory response by modulating leukocyte chemotaxis and inhibiting the production of pro-inflammatory mediators such as cytokines, which in turn may facilitate bacterial immune evasion (14). Mutagenesis of S5nA in three phylogenetically distinct GAS strains however had no effect on host survival in a *Galleria mellonella* model of infection (15). A major caveat in using insect models to study human disease is the absence of an adaptive immune system, which can be regulated by extracellular adenosine in mammals (5). By stimulating A_2A_ receptors, adenosine can inhibit adaptive immunity and limit inflammation by restricting T cell activation and effector function. It has been recently shown that an ectonucleotidase of *Staphylococcus aureus*, an invasive bacterial pathogen like GAS, can generate adenosine to stimulate A_2A_ receptors and subsequently restrain T helper 17 cell immunity and promote recurrent infection (16). Thus, it can be hypothesised that a similar strategy may be used by GAS through the secretion of S5nA to evade host adaptive immunity (**Figure 1**), which could be assessed using more complex systems such as murine models (17) or humanised mouse models (18) of GAS infection.

In addition to adenosine generation and subsequent adenosine receptor activation, bacterial ectonucleotidases may limit immunity to GAS by reducing the concentration of extracellular nucleotides and limiting the activation of pro-inflammatory P2 receptors. ADP, a substrate of S5nA, is a natural agonist of P2Y_1,12,13_ with roles in haemostasis and immune cell regulation. Knockout of P2Y_1_ in mice decreased their survival in a model of *Pseudomonas aeruginosa* infection (19) while activation of P2Y_12_ and P2Y_13_ enhanced macrophage-mediated immunity in a murine model of *Escherichia coli* infection (20). As such, ADP hydrolysis by S5nA may increase GAS survival by dysregulating P2 receptor-mediated inflammatory processes including platelet activation, macrophage or neutrophil recruitment, or other processes prominent in GAS pathology, such as wound healing (21) in which P2 receptor activation is involved (22, 23). The broader effects of this may include activation or inhibition of intracellular signaling pathways and components downstream of purinergic receptor activation, such as inflammasomes, mTOR and mitochondria.

## Emerging Roles of P2X7 in GAS Infection

Due to its role in cytokine production, T cell activation, and cell survival, P2X7 is the most studied P2 receptor in relation to host immunity against bacterial infections (24). P2X7 is abundantly expressed on immune cells including neutrophils and macrophages (24), which are considered the chief regulators of GAS immunity (25). Activation of P2X7 by extracellular ATP triggers the assembly of the NOD-like receptor family pyrin domain-containing 3 (NLRP3) inflammasome that activates caspases to stimulate pro-inflammatory interleukin (IL)-1β maturation and release (26). Since excessive cytokine release and hyperinflammation has been associated with both P2X7 activation (24) and invasive GAS pathologies (10), several studies have investigated a role for P2X7 in GAS infection.

Virulence factors of GAS have been shown to evade P2X7 activation and function. The endogenous immune cell peptide, cathelicidin LL-37, is a potent antimicrobial and chemotactic agent (27) and can activate P2X7 in the absence of extracellular ATP (28). LL-37 may therefore represent a non-nucleotide P2X7 agonist but its mechanism of action remains unclear (29). Of note, secretion of a GAS protease, ScpC, cleaves LL-37 into fragments to prevent P2X7 activation and reduce neutrophil chemotaxis and longevity *in vitro* (30) (**Figure 1**). Moreover, in a murine model of deep tissue infection, ScpC-knockout GAS increased neutrophil recruitment and prevented dissemination into deeper tissue, a protective phenotype that was abolished using wild-type GAS (30). P2X7 can also promote the internalisation of LL-37 to enhance the killing and clearance of intracellular *S. aureus* in human macrophages (31) but whether this process also occurs for GAS remains unknown. Similarly, a role for P2X7 in GAS infection has been demonstrated through another secreted virulence factor, β-nicotinamide adenine dinucleotide^+^-glycohydrolase (NADase). NADase can inhibit P2X7-mediated IL-1β release from human and murine macrophages challenged with M1-expressing GAS (32). Together, these studies suggest important roles for P2X7 in protective host immunity against GAS infection. In contrast to ScpC and NADase, activation of P2X7 protects human mast cells from streptolysin O (SLO)-induced cytotoxicity by initiating membrane blebbing as a mode of cell repair in response to GAS (33). However, SLO can induce human and murine macrophage NLRP3 inflammasome activation and IL-1β release independently of P2X7 activation (34).

Collectively, the above studies indicate that the role of P2X7 in GAS infection varies between host cell types. Moreover, other GAS virulence factors, such as M protein and SpyA, can also induce NLRP3 inflammasome activation and IL-1β release in macrophages (35, 36), and GAS infection can induce IL-1β release from neutrophils (37, 38) but a role for P2X7 in these settings has not been investigated.

## Future Directions and Therapeutic Potential of Purinergic Signalling in the Resolution of GAS Infections

Current treatments to resolve GAS infections are limited to antibiotics, intravenous immunoglobulin therapy or surgical intervention (3). The number of antibiotics approved by Food and Drug Administration has dramatically declined since the 1980’s with β-lactam antibiotics such as penicillin remaining the primary course of treatment for GAS clearance (39). While there is no report of penicillin resistance in GAS to date, antibiotic resistance has become one of the most significant health issues of the 21^st^ century (40). Coupled with GAS treatment failure (41), this has focused research on non-antibiotic therapeutic targets such as purinergic receptors and their ligands.

Understanding the molecular mechanisms underlying invasive GAS pathogenesis and host persistence are a prerequisite for novel vaccine and therapeutic developments that can prevent recurrent infection and disease progression. Although a vaccine has yet to be approved, the GAS virulence factors S5nA and ScpC, which are highly conserved, have been proposed as potential vaccine candidates (42). Immunisation of mice using S5nA and ScpC antigens increased host survival across different GAS serotypes, inclusive of the clinically relevant M1-expressing GAS infection (42) supporting their potential as vaccine targets. Notably as described above, both virulence factors are linked to host purinergic signalling pathways, suggesting that such vaccines may promote antibodies that can neutralise S5nA and ScpC to prevent unwanted modulation of purinergic signalling.

The notion of purinergic receptors as therapeutic targets has been demonstrated in a variety of pre-clinical applications to date (43), however limited progress has been made in the context of GAS infection. Patients receiving Anakinra, an IL-1 receptor antagonist, were documented to have an alarmingly increased susceptibility to developing necrotising fasciitis, a severe soft tissue infection of GAS, with an associated high mortality rate (44). This finding supports a vital role for IL-1β release in controlling GAS infection. Given that the contribution of P2X7 may be minor compared to other mechanisms of IL-1β release in GAS infection (32, 34), coupled with the protective roles of P2X7 in GAS immunity (30, 32, 33), potentiating P2X7 activation with therapeutic agonists may safely reduce GAS hyperinflammation and disease severity without exacerbating pro-inflammatory cytokine production.

Finally, other purinergic receptors, particularly P2X4, may also be important in regulating GAS infection. A recent study observed that P2X4 activation was integral for macrophage-mediated immunity in a cecal ligation and puncture sepsis model (45). Specifically, activation of P2X4 by ATP decreased bacterial load and increased inflammatory cytokines to enhance murine survival. Notably, selective treatment with ivermectin, a positive modulator of P2X4, showed analogous protective effects in mice but without increasing inflammatory cytokine production (45). Given that GAS-induced sepsis is often accompanied by aberrant cytokine release (10), selective modulation of P2X4 may improve host survival and offer novel therapeutic direction.

Given the current knowledge of GAS pathogenesis, and the diverse functions of the purinergic signalling axis, harnessing purinergic ligands or receptors as therapeutic targets may lead to new developments in the clinical space to limit the severity of GAS infections.

## Author Contributions

All authors have read and approved the final manuscript. TM (Conceptualisation, visualisation, writing – original draft); MS-S (Conceptualisation, validation, supervision, writing – review and editing); RS (Conceptualisation, validation, supervision, writing – review and editing).

## Funding

TM is supported through an Australian Government Research Training Program Scholarship. MS-S and RS are supported by Molecular Horizons and a “UOW Near Miss Grant” from the University of Wollongong.

## Conflict of Interest

The authors declare that the research was conducted in the absence of any commercial or financial relationships that could be construed as a potential conflict of interest.

## Publisher’s Note

All claims expressed in this article are solely those of the authors and do not necessarily represent those of their affiliated organizations, or those of the publisher, the editors and the reviewers. Any product that may be evaluated in this article, or claim that may be made by its manufacturer, is not guaranteed or endorsed by the publisher.

## Abbreviations

ADP, adenosine 5’-diphosphate; AMP, adenosine 5’-monophosphate; ATP, adenosine 5′-triphosphate; GAS, Group A *Streptococcus*; IL, interleukin; NADase, β-nicotinamide adenine dinucleotide^+^ glycohydrolase; NLRP3, NOD-like receptor family pyrin domain-containing 3; S5nA, *streptococcal* 5′-nucleotidase A; SLO, streptolysin O.
